# BabelFSH—a toolkit for an effective HL7 FHIR-based terminology provision

**DOI:** 10.1186/s13326-025-00343-4

**Published:** 2025-11-29

**Authors:** Joshua Wiedekopf, Tessa Ohlsen, Ann-Kristin Kock-Schoppenhauer, Josef Ingenerf

**Affiliations:** 1 Institute of Medical Biometry and Statistics, Section for Clinical Research IT, University of Luebeck and University Hospital Schleswig-Holstein, 160 Ratzeburger Allee, 23562 Luebeck, Germany; 2 Institute of Medical Informatics, University of Luebeck, Luebeck, Germany

**Keywords:** HL7 FHIR, Terminology as topic, Terminology servers, Knowledge bases

## Abstract

**Background:**

HL7 FHIR terminological services (TS) are a valuable tool towards better healthcare interoperability, but require representations of terminologies using FHIR resources to provide their services. As most terminologies are not natively distributed using FHIR resources, converters are needed. Large-scale FHIR projects, especially those with a national or even an international scope, define enormous numbers of value sets and reference many large and complex code systems, which must be regularly updated in TS and other systems. This necessitates a flexible, scalable and efficient provision of these artifacts. This work aims to develop a comprehensive, extensible and accessible toolkit for FHIR terminology conversion, making it possible for terminology authors, FHIR profilers and other actors to provide standardized TS for large-scale terminological artifacts.

**Implementation:**

Based on the prevalent HL7 FHIR Shorthand (FSH) specification, a converter toolkit, called *BabelFSH*, was created that utilizes an adaptable plugin architecture to separate the definition of content from that of the needed declarative metadata. The development process was guided by formalized design goals.

**Results:**

All eight design goals were addressed by BabelFSH. Validation of the systems’ performance and completeness was exemplarily demonstrated using Alpha-ID-SE, an important terminology used for diagnosis coding especially of rare diseases within Germany. The tool is now used extensively within the content delivery pipeline for a central FHIR TS with a national scope within the German Medical Informatics Initiative and Network University Medicine and demonstrates adequate usability for FHIR developers.

**Discussion:**

The first development focus was geared towards the requirements of the central research FHIR TS for the federated FHIR infrastructure in Germany, and has proven to be very useful towards that goal. Opportunities for further improvement were identified in the validation process especially, as the validation messages are currently imprecise at times. The design of the application lends itself to the implementation of further use cases, such as direct connectivity to legacy systems for catalog conversion to FHIR.

**Conclusions:**

The developed *BabelFSH* tool is a novel, powerful and open-source approach to making heterogenous sources of terminological knowledge accessible as FHIR resources, thus aiding semantic interoperability in healthcare in general.

## Background

### HL7 FHIR for national and international standardization and harmonization

Both national and international demands on healthcare data interchange have made it ever-more important for healthcare providers to cooperate and to provide their primary-care data in machine-readable formats. Legislation in many healthcare systems, such as the US *21st Century Cures Act* passed in 2016 [1, 2], or the emerging *European Health Data Space* (EHDS) [3] in the European Union, have catalyzed this requirement. Concurrently, national initiatives in the research domain, such as the German *Medical Informatics Initiative* (MII) have accelerated this need for interchange and alignment to common standards even further [4].

Such large-scale integrations between disparate systems require harmonization and standardization, especially towards common data structures, encodings and processes. The use of interoperability standards presents itself as a suitable approach in this regard, and the *Health Level 7 Fast Healthcare Interoperability Resources* Standard (HL7 FHIR standard) [5] has been recognized to be a suitable means to this end in many jurisdictions [6, 7].

HL7 FHIR is inherently designed as an internationally applicable standard to support the broadly different requirements of different jurisdictions. As part of this design requirement, extensibility and adaptability were dominant considerations in the standard development processes. Consequently, national actors as well as use-case driven consortia are expected to define and utilize FHIR profiles, derived from the core standard [5, Sect. 5.1.0], to tailor the definitions of the standard to their needs.

### Profiling FHIR and valueset bindings

Apart from constraining element cardinality and adding extensions where applicable, the profiling process also generally changes numerous terminology bindings for coded data elements [5, Sect. 5.1.0.19]. As a rule, all coded data elements in FHIR must be bound to a *ValueSet*, which in turn is defined as a subset of codes from one or more code systems. All coded data elements in the core FHIR standard have bindings already applied, but profiling often overrides these to better fit the reality of the respective jurisdiction. This is a core mechanism in the FHIR philosophy.

From this large need to define bindings in profiles arises a need to provide *ValueSet* resources to technical consumers. *ValueSet* (VS) resources in FHIR pick codes from one or more code systems. The standard also defines the resource type *CodeSystem* (CS) for the representation of these definitions within FHIR resources. In view of the enormous heterogeneity and complexity of classifications (e.g. ICD-10 or ATC with their respective national adaptions) and terminologies (e.g. SNOMED CT or LOINC), their conversion into FHIR code systems can be at times quite challenging and is the main subject of this paper. To facilitate mappings between code systems, e.g. from non-standard local towards standard international terminology, *ConceptMap* (CM) resources can also define unidirectional mappings scoped to a certain use case. To implement system interactions with these resources, FHIR terminological services (TS) have emerged from a subsection of the specification [5, Sect. 4.0] that provide both read-write access to FHIR resources and allow operations-based interactions with these resources.

### FHIR representation of terminological concepts

In this paper, a careful differentiation is made between the intellectual concepts of *code systems*, *value sets*, and *concept maps/mappings* and their manifestation in FHIR through the *CodeSystem* (CS), *ValueSet* (VS) and *ConceptMap* (CM) resources. The first group of concepts has been defined in the HL7 Version 3 Core Principles [8] and have been carried forward into the design of the FHIR standard. It is stated there that “*Code System*s are often described as collections of uniquely identifiable concepts with associated representations, designations, associations, and meanings” [8, Sect. 5.1.2]. Value sets are then defined as “[representing] a uniquely identifiable set of valid concept identifiers, where any concept identifier in a coded element can be tested to determine whether it is a member of the *Value Set* at a specific point in time.” [8, Sect. 5.1.3]. Concept mappings were not in-scope for the standard at this time and were introduced in the FHIR DSTU1 release.

However, the HL7 Version 3 standard, and early FHIR versions including the DSTU2 release, did not provide a representation (via a resource type in FHIR) for representing the content of a code system itself. Instead it was understood that complex, standard terminology will be managed through independent means. The DSTU2 release of FHIR states the following:

Value sets that contain **inline code systems are intended for small**,** simple code systems** that are found throughout the implementation context (e.g., lists of words, status codes, enumerations). The inline code system definition is **not intended to represent large publically defined terminologies** such as LOINC, etc. - **these terminologies have their own distribution formats**. [9, Sect. 6.21.1; our emphasis]

As such, small code systems were manifested through their use within *ValueSet* resources, providing definitions for small, constrained applications such as communicating the status of an *Observation* resource. Larger standard terminologies were always understood to be maintained and distributed independently. For consuming and producing systems that communicate using these codes, it was then assumed that they would always have access to the required terminology to then expose it to their users and internal processes. Consequently, terminology developers have developed extensive editorial guidelines [10] and tools [11, 12] for the maintenance of their resources to best capture their development processes.

In FHIR STU3, it was realized that many terminological use cases could be addressed by representing code systems in a FHIR resource of their own. The in-line definition in the VS resource worked well for constrained use cases, where the value set is used just a few times within the standard. However, this approach does not scale with the representation of standard terminology, which generally is intended to be used in many different use cases and value sets. While the then-created *CodeSystem* resource was not supposed to be used for the maintenance process of most terminology, which was still understood to be better served by the established processes [5, Sect. 4.8.1], most coding systems can thus be expressed using native FHIR resources. This allows for the definition of FHIR-based terminology services for code systems that are not considered internal to the HL7 specification and derived artifacts, but also large code systems brought into the server through their FHIR representation.

**Fig. 1:**
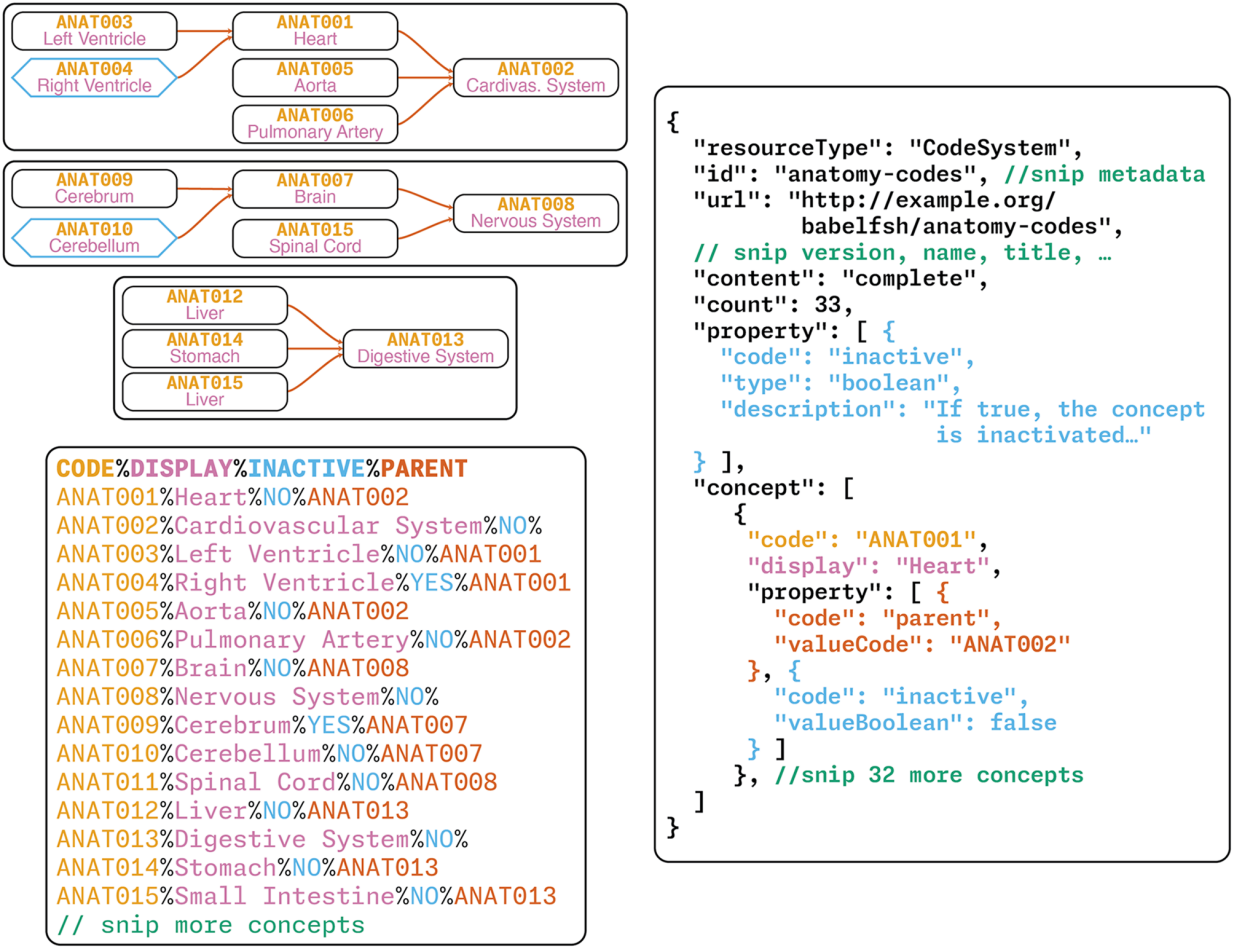
Representation of terminology resources through arbitrary formats. Top left: graph representation of concepts and hierarchies. Bottom left: plain-text source file in a CSV-style format Right: FHIR JSON representation derived from the CSV input. This resource was provided in the evaluation process of BabelFSH for conversion into a FHIR CodeSystem, with the content of this resource was generated by a large language model (Claude Sonnet 4). The colors illustrate correspondences between the concepts. Teal diamonds in the graph represent inactivated concepts

To differentiate between these abstract concepts and their manifestations in FHIR, in this paper the long forms *code system*, *value set*, and *concept mapping* will only be used to reference the abstract concepts and resources that are not represented in FHIR resources. The forms *CodeSystem*/*ValueSet*/*ConceptMap* and the abbreviations *CS*/*VS*/*CM* are only used to reference the FHIR manifestation. The boundary between the intellectual concept of a code system, the representation in a suitable domain-specific format and the representation in FHIR is illustrated in Fig. 1.

As is common in the HL7 FHIR standard’s design, the CS resource can only capture a subset of use cases directly. Based on the 80/20 rule, FHIR resource development should be “focus[ed] on the 20% of requirements that satisfy 80% of the interoperability needs” [5, Sect. 2.1.19.2], leading to limitations in the expressivity of some resources for specialized use cases.

For example, formal ontologies that are often expressed in the Web Ontology Language (OWL) often build on top of each other. The OBO foundry, a project that aims to develop a number of interoperable biomedical ontologies, states in their guiding principles that the “scope” of any ontology they oversee “should be fairly narrow” and that “[r]equired terms that are out of scope should be imported from the appropriate ontology unless no such ontology exists” [13]. For example, a narrowly-scoped ontology in the domain of human disorders can build on the concepts defined in an ontology describing cross-species anatomy in the defining axioms [14]. The narrowly scoped concepts (*classes* in the convention of formal ontologies) are then defined as sub-classes or children of those imported classes. The subsumption relationship in the domain of formal ontologies has a very rigid definition [15]. Every subclass of a class is also an instance of the parent class, leading to the strict, transitive, *is-a* relationship. In this way, connected ontologies span a large semantic network through their *is-a* relationships. While this relationship can be expressed in the CS resource, using custom properties that link concepts across CS boundaries, the subsumption relationship in FHIR TS is only defined within the scope of a single CS. Consequently, a standard implementation would not be able to evaluate the subsumption relationship between those concepts correctly. Regardless, a conversion of the code systems defined in formal ontologies to the FHIR CS resource can be beneficial, as highlighted by Metke-Jimenez et al. [14].

Another shortcoming of the CS resource in particular is the impossibility of representing compositional code systems, such as the Unified Code of Units and Measures (UCUM) [16]. Units in UCUM can be combined arbitrarily, to express measurements in any kind of physical dimension. The formal grammar allows an infinite number of combinations of the defined unit symbols, especially in conjunction with the annotation facilities that can be used to express countable concepts. For example, the expression *{FHIR CodeSystem resources}/[nmi_i].s2* (countable things per nautical-mile per second-squared) is non-sensical, but valid in the language defined by the UCUM grammar. While a finite fragment of available and common UCUM codes could be created and distributed, the FHIR CS resource lacks the means to express the grammar-based UCUM standard. A FHIR TS could, however, provide some interactions defined in the terminology module of the standard through special casing, such that validation of UCUM codes can be implemented adequately.

### Terminological services within national projects

Terminological services are an important factor towards healthcare data interoperability and, ultimately, data harmonization. The German MII (since 2015) and Network University Medicine (NUM, since 2020), funded by the German Federal Ministry of Research, Technology and Space, have recognized this need. In this funding scheme, the sub-project *Service Unit Terminological Services* (SU-TermServ) [17] has been tasked with providing terminological services through a FHIR terminology server, beginning in 2023. The required content is, in particular, defined and specified by the modular Core Data Set (CDS) of the MII [18]. From the Germany-wide drive to implement and exchange CDS-compliant FHIR resources, the need for a harmonized tooling to provide FHIR-based representations of referenced code systems originated. The core of the SU-TermServ project revolves around the provisioning of a central instance of Ontoserver [19] with all needed terminology resources [20]. In this way, the project’s requirements are driven by the CDS dataset definition, which utilizes a broad array of national and international terminology resources. To provide the required FHIR operations, these resources must be available through the respective FHIR resource types. For the professional support of a TS with such a national scope, transparent and effective processes must be established to provide the needed resources at the correct time for the TS to then generate a benefit for the surrounding health technology landscape.

This is at odds with the current reality, whereby most well-recognized code systems are instead maintained using specific tools and platforms and are often provided using proprietary formats or using very different standards. For example, the ICD-10-GM resource (the German national adaptation of the international diagnoses classification) is maintained by the German Federal Institute for Drugs and Medical Devices (*BfArM*) using internal processes, taking change requests from the medical community into account. As of writing, the document-based distribution in PDF format is the authoritative version, but a number of machine-readable formats are made available: one version uses the ISO ClaML standard (Classification Markup Language, ISO 13120) [21], while one format uses tab-separated files for import into relational databases^1^.

On the other hand, OncoTree, a classification for tumor types [22] is distributed mainly through an unstandardized web-based application programming interface (API). Considering also the fact that internal catalogs are often only available directly in the systems they are defined within, a clear need for simple and extensible toolkit for conversion of terminological artifacts into the HL7 FHIR resources *CodeSystem*, *ValueSet* and *ConceptMap* emerges. In Table 1, a brief survey of some terminologies referenced within the CDS of the MII further illustrates this need.

**Table 1:** Exemplary terminologies/code systems referenced in the German Medical Informatics Initiative’s Core Data Set with distribution formats

Name	Use Case	Distribution Format
**Diagnosis coding**		
ICD-10-GM	German adaptation of the ICD-10 for morbidity coding	ClaML, **some versions as FHIR CS**
ICD-10-WHO	Mortality coding	ClaML, **some versions as FHIR CS**
ICD-O-3	Cancer classification	ClaML
Alpha-ID-SE	Coding for rare diseases aligned to ICD-10-GM	Columnar text file
ORPHAcodes	Coding for rare diseases	XML
OncoTree	Cancer classification	Web API
**Procedure coding**		
OPS	Procedure coding	ClaML
**Medication coding**		
ATC (German & International variant)	Pharmaceutical agents	Spreadsheet (Office Open XML *XLSX* format)
ASK	Pharmaceutical agents (on the substance level)	XML
CAS	Chemical substances	Partially available in the ASK XML file
EDQM Standard Terms	Pharmaceutical dose forms, routes of administration, …	Web API
UNII	Pharmaceutical agents (on the substance level)	Columnar text file
PZN	Commercially available pharmaceutical products in Germany	Columnar text file
**Medical Devices & Imaging**		
ISO/IEEE 11073-10101	Medical device communication standard terminology	**FHIR CS**
DICOM DCM	Medical imaging standard	**FHIR CS**
RadLex	Radiological lexicon for reporting	OWL
**OMICS data**		
HGNC	Gene symbols/names	Columnar text file or JSON
GENO	Gene functions	OWL
HPO	Phenotypes	OWL
SO	Sequence annotation	OWL

As outlined above, the FHIR standard has always anticipated this fact, but many FHIR-based terminology services require FHIR-based representations of code systems to provide the resources through the APIs defined in the terminology module. A FHIR TS, which is specified in a functional manner through its API [5, Sect. 4.7], is not required to support *create-read-update-delete* operations for terminology resources, such that the implementation of a FHIR façade as the interface to a proprietary database is standards-compliant. However, the alternative approach, whereby the terminology is directly maintained by the system developer in proprietary formats and included in the binary distribution, is often not suitable for large-scale projects such as the MII and NUM. As those projects inherently move fast, the resulting dependency on the TS developer to provide new terminologies in an agile fashion can be a limiting factor in the projects’ development.

Without the terminology artifacts required by the respective domain at the correct time, even best-of-breed terminology services cannot generate a benefit to the surrounding landscape and can thus be considered of no use. This work thus proposes a novel solution for making terminologies available through FHIR resources, building on established standards and allowing for rapid implementation for highly specific conversion processes. By bringing the resources to the FHIR standard, terminology maintainers are empowered to distribute FHIR representations of their resources within their editorial and technical pipelines. In this way, the proposed tool strengthens the semantic component of the FHIR standard by ensuring that relevant terminology resources are accessible to the ecosystem.

### Prior work

The research community has so far not addressed the problem of terminology generation for HL7 FHIR extensively. The developers of *Ontoserver*, a FHIR-based terminology server in widespread use and the basis for the service provided by the SU-TermServ, have touched upon the generation of resources in their 2018 paper discussing the software [19]. This includes support for ClaML [21] generation using the *ClaML-FHIR* tool [23]. From the same working group, a transformation of Web Ontology Language (OWL) ontologies into FHIR was presented in 2019 [14].

Moreover, a literature review reveals several transformation scripts for specific resources. The authors have for example provided scripts for OncoTree and the EDQM Standard Terms database [22, 24–26], and have discussed further challenges in CS and VS transformation from the ART-DECOR platform, already touching upon the problems outlined above [27].

The authors of the *TerminoloGit* terminology server [28] have integrated a tool called *MaLaC-CT* into their pipelines, which converts the between all formats required by the Austrian national healthcare system. However, the scope of this tool is limited to the supported input and output formats. For transforming resources that don’t conform to one of the supported input formats, a secondary conversion step would be needed.

Besides automatic conversion scripts and tools, FHIR-based terminology editors like *TermX* [29] or *Snapper* [30] (part of the Ontoserver [19] development effort) have been put forward. Both tools can be used to directly create resources via the web interface and allow for importing external sources via tabular files. These files, delimited by commas, tab stops or semicolons, can be created using spreadsheet applications, and are generally referred to as CSV (*Comma Separated Values*) formats. While this format is very accessible to non-technical domain experts, it is inherently records-oriented and limited in its expressivity. For example, for expressing multiple properties with the same code for a concept in a FHIR CodeSystem, Snapper can merge rows with the same code into one record, while TermX raises an error for the same approach, as it uses internal delimiters in the columns for repeating properties. Hence, for the primary maintenance of domain-specific resources, both tools require standardized operating procedures to ensure compatibility of the spreadsheet data with the system. While TermX also supports importers for specialized terminologies and can be extended with custom import adapters due to its open-source nature, it is intended to be an end-to-end solution for terminology maintenance. As such, it supports a concept-oriented view that tracks changes to concepts over time, assigning tasks for curation of the terminologies, wiki pages, and much more. Snapper is much more limited in scope, and exhibits performance problems with large terminologies, since it keeps all data in browser memory rather than in a performant backend. As such, it is only usable for simple resources. TermX, meanwhile, offers a powerful and very user-friendly approach for primary creation of terminologies that can offer benefits to terminology maintainers. For established terminologies, it requires the implementation of specific adapters to support their data formats.

Especially considering the open-source code of the identified conversion tools, a key insight towards simplifying this process is the fact that most tools follow a similar structure. First, the metadata of the resources, e.g. the *canonical URI*, the *version*, *description*, *author*, *copyright*, and related fields are generated. Sometimes, this is hard coded within the tool, while in other instances, these data are defined using configuration files or provided as command line arguments.

Once the metadata of the resource is defined, the tools then take in the authoritative sources of the artifact and generate the needed content: for a CS, a list of *concept* elements, for VS, *include* and *exclude* elements, and for a CM, a list of *groups*, which in turn contain *elements*. The content generator functionality in the tools can be highly specific (e.g. for OncoTree) or could also be generalizable to many different resources using the same formats (e.g. for ClaML).

Finally, the resources are written to disk. While the implementation differs broadly, this pattern of defining metadata first and generating content afterwards holds for all related tools thus identified.

Another insight from the literature are the degrees of freedom associated with providing FHIR resources from other sources. For CS, consider the representation of hierarchical relationships: *concept* elements can define *concept* elements in-line, representing a strictly monohierarchical relationship. The representation through a property, generally using the *parent* and/or *child* relationship code, is however anecdotally preferred by TS implementers; and the FHIR standard states that they should not be used concurrently [5, Sect. 4.8.13]. However, one CS might only use *parent*, another only *child*, and another one might link concepts in both directions with both properties.

In rare cases, resources might not use those standard properties, instead assigning a different kind of relationship between concepts. The rare disease nomenclature ORPHAcodes [31], defined by the Orphanet database [32], for example, uses the concept of *aggregation levels* to link *disorders* and *subtypes of disorders*. Depending on the reporting use case, more specific or more general concepts might be required. This property can be understood as a special case of parent-child relationships, yet there is not a single parent for each property as in many other classifications. Instead, the terminology defines a “forest” of small “trees” through the aggregation level. In some use cases, such as feasibility queries [33], this relationship can be considered to be equivalent to the parent/child relationships, as researchers selecting a single concept from the user interface also reasonably expect the subtypes of that disorder to be selected. This needs to be achieved either by the search interface providing special consideration for the code system, by the TS interpreting this relationship as equivalent to the parent/child relationship, or by the FHIR resource serializing this property via the standard parent/child relationship.

### Objective

The aim of this work is to provide to the broader FHIR community a simple-to-use and simple-to-extend toolkit for providing computable terminological resources for terminologies, classifications, ontologies, etc. as well as value sets and concept mappings, derived from their native distribution formats, building on established mechanisms. Ultimately, the implementation of such a tool is intended to comprehensively support FHIR implementers and standard development organizations who are responsible for the first-party maintenance of terminologies to provide them with the opportunity to provide these resources themselves. In this way, this work is a step towards providing strong semantic support to the FHIR standard.

The key architectural principle derived from the literature is the clear separation between the definition of resource metadata and the generation of content. Metadata generation refers both to metadata that describes the resources in their entirety, such as name, ID, canonical URI etc., and to metadata that can be applied to CS concepts, i.e. *properties*. The metadata generation should follow a uniform approach across all resources, thus allowing for harmonization and convention across resources. In contrast, content generation must support both reusable, generic methods and highly specific transformations, such that all requirements of the terminologies in question can be addressed appropriately.

Through this tool, the representation of terminology resources can be harmonized through the re-use of rules, conventions and transformation logic, thus aiding terminology service adoption.

## Implementation

### Architecture

The FHIR Shorthand (FSH) language specification [34] has quickly become a cornerstone of the FHIR community, and is now considered the dominant means to define FHIR resources as source-code using a domain-specific language (DSL). By compiling the source code files with the reference compiler, SUSHI [35], the definition of profiles and other *StructureDefinition* resources has been simplified and streamlined.

FSH’s language elements are geared towards the concise definition of FHIR resources, especially profiles and extensions, but also resource instances of all other resource types, such as example resources within an *ImplementationGuide* (IG). Crucially, the FSH language and the SUSHI compiler also support the definition of *CodeSystem* and *ValueSet* resources directly, and *ConceptMap* via the arbitrary-resource facilities. However, especially the CS support is geared towards the definition of small, internal CS with only a few codes. All concepts must be listed in-line of the entire FSH source file (see an example in Fig. 5). Generating FSH sources from original content and then compiling this with SUSHI is in our experiments certainly possible, but computationally quite expensive.

However, as SUSHI is the reference implementation of an open-source formal language specification, and only a subset of this specification is relevant to the terminology generation process, the implementation of a use-case specific compiler tool was initiated. The developed open-source tool is called *BabelFSH*.

The tool is built using the Gradle build automation toolkit [36] in a modular build with dependencies between these modules. Its implementation uses the Kotlin programming language [37], utilizing the Java Virtual Machine.

**Fig. 2:**
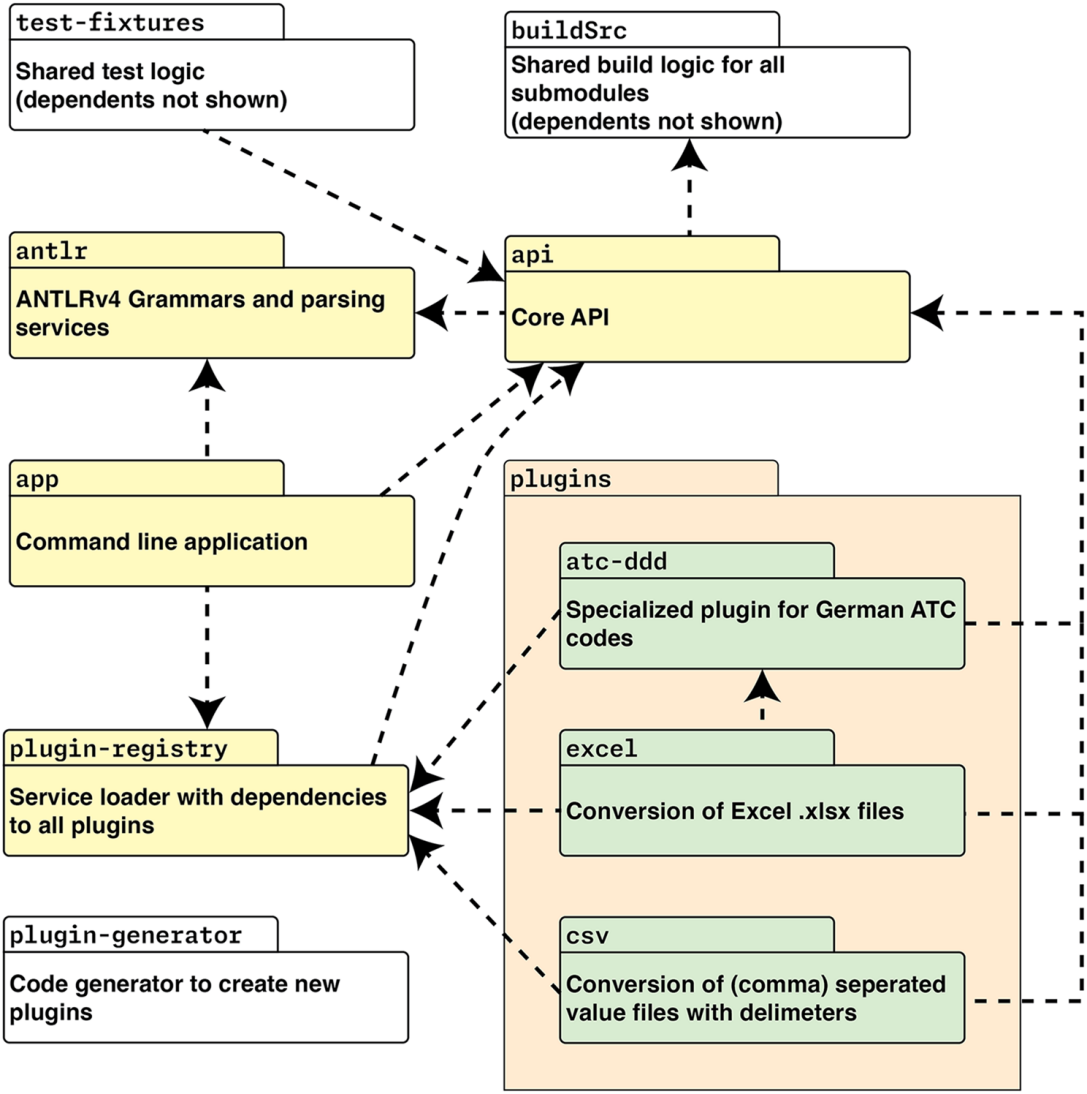
High-level architecture of the BabelFSH system, showing Gradle modules and their interaction in the overall system. Yellow modules are the application’s foundation, while white modules are mostly required for development of the application. Only a selection of plugin modules is shown due to space constraints

The high-level architecture of the developed system is shown in Fig. 2.

### Design goals

Prior to the implementation of the system, eight design goals that the tool must adequately address have been defined:

- **DG1—Compatibility with SUSHI**: The BabelFSH source files shall remain completely compatible with the SUSHI reference implementation (only) for *CodeSystem*, *ValueSet* and *ConceptMap*. No language elements shall be added to FSH DSL that result in errors when using SUSHI to compile BabelFSH source files. Thus, changes in the FSH specification going forward can be incorporated into BabelFSH without delay.
- **DG2—Metadata in FSH**: The FSH DSL shall be used to define the metadata of the resulting terminology resources. The core FSH language elements shall not be used to define the concepts of CodeSystem resources. This requirement enforces the beneficial separation of metadata and content that was identified from the literature.
- **DG3—Extensibility**: It shall be easy to generate content from diverse sources. Core functionality shall be provided to implementers to address common requirements. If a code system requires highly specialized logic, this must be supported. Otherwise, the expressivity of the system would be so limited that many resources would not be convertible, dramatically limiting the usefulness of the system.
- **DG4—Simple API and Self-Documentation**: Implementers shall be provided a simple API to hook into the generation process, such that needed logic can be implemented quickly, allowing for fast turnaround and easy maintenance.
- **DG5—Validation**: Generated resources shall be validated for their correctness against the FHIR specification. Especially syntactic problems shall not be accepted, but semantic validation should also be performed if applicable. A strong validation process helps to ensure consistent resources, aiding implementers in creating correct and comprehensive metadata for their resources.
- **DG6—Performance**: BabelFSH shall be performant, such that the generation of multiple versions of large code systems can be carried out without issue and should offer substantial performance benefits over native FSH compilation. Were the performance of BabelFSH similar to SUSHI, the benefit of a separate parser/compiler would be offset by the complexity of this tool.
- **DG7—FHIR Version support**: The tool shall support multiple versions of the FHIR core specification. At the time of writing, this especially requires support for versions R4/R4B and R5, leading to a need of special consideration especially in the CM generation aspect, since CM has undergone considerable conceptual changes in FHIR R5. The user must be explicit in which FHIR version they use, the concurrent generation of R4B and R5 resource in the same application invocation shall not be supported. This ensures relevance of BabelFSH going forward, such that later versions of FHIR can also be integrated.
- **DG8—Open Source**: BabelFSH must be licensed with an open-source license, so that the FHIR community can, without any restrictions for commercial use, make use of the tool, so that enhancements can also be fed back into the applications. Due to the open nature of the FHIR community, the provision of an open-source tool can foster collaboration and improve the adoption of FHIR terminology services and FHIR in general.

With respect to **DG2**, *metadata* are defined as those data elements available in terminological resources to describe the resource in its entirety (i.e., the canonical URI, version, name, title, technical ID, publisher, etc., as well as extensions for the entire resource), and also those data elements to describe the concepts defined within a CS (i.e., properties). The first kind of metadata is defined by the FHIR core standard, and FSH rules will be required to fill the respective slots.

Properties follow a code-value-paradigm, whereby the declaration assigns each property a code and data type. The FHIR specification implicitly defines a selection of properties that need not be declared in CS, such as the *parent*, *child*, or *inactive* properties that are commonly used in FHIR CS resources. All other properties must be declared in the FSH source and will then be used in the plugin implementation when concepts are generated. General converter implementations **(DG3)** then need to provide configuration options to map properties in the terminology sources to the properties generated by the plugin.

Concerning **DG5** and **DG7**, supporting either R4B or R4 is adequate, as no changes between these versions affect the terminology module [5, Sect. 2.1.11], and validation using either of these versions will result in the same output messages.

### Parsing FSH code

To parse the FSH source files needed for the BabelFSH system, the free and open-source parser generator ANTLR v4 [38] is used. A comprehensive overview of the parsing algorithm is available in the software’s documentation. The grammar is based on the implementation for the FSH reference implementation, SUSHI [35], which also utilizes ANTLR v4. By modifying select sections of the SUSHI grammar and adding other grammars as needed, parseable language elements were added to the generated FSH parsers.

To remain compatible with standard FSH code (**DG1**), the implementation uses block comments, which are an existing language element of FSH, to define command line arguments to the plugins. Block comments follow the C-style syntax using a slash and asterisk symbol at the leading and trailing end of the comment. For these comments to be considered by BabelFSH, a special recognition token (leading: /*^babelfsh, trailing ^babelfsh*/) was introduced, so that normal comments are not hijacked by this approach. Errors are caught early in the processing and are surfaced to the user in meaningful error messages, pointing them towards the line where a declaration does not follow the FSH language specification.

### Application programming interface

To allow for easy extensibility (**DG3**) of the core logic of the application, a plugin-based approach has been implemented. Through the modular build, plugins are maintained in sub-modules of the overall application. They are discovered and registered automatically and are identified using a unique plugin ID, and need to define the arguments they expect, such as input file paths or URIs, columns to map, or properties to render. To make the declaration of these arguments in the FSH code simple and understandable (**DG4**), an established command line parsing library for Kotlin is used [39]. Plugins then declare short and long forms of their needed arguments, such as --file/-f for the input file, and associated help texts for each argument. By declaring optional and required arguments in the source code, validations for the provided argument values, and the facility to validate the interaction between e.g. mutually exclusive arguments, the plugins receive a type-safe set of arguments they can use in concept generation. As plugin developers also need to provide help texts for the arguments, BabelFSH can automatically generate an interactive help, and can give the user detailed feedback on incorrect argument use without effort to the plugin developer (**DG4**). Based on these help texts, online documentation can be pre-generated, such that additional information can be provided to users.

The plugins are intended only for content generation, not the definition of metadata, in line with **DG2**. Abstract classes define the entry points for this content generation:

- For CS resources, the generation of concept entries,
- For VS, the definition in terms of inclusions and exclusions,
- And for CM, the definition of groups.

Each plugin must implement only two methods, one for parsing the command-line arguments in a type-safe fashion, one for generating the content.

To support multiple FHIR versions in the same application **(DG7)**, the plugins do not natively generate FHIR resources. Instead, they use version-agnostic proxy classes, which are serialized as FHIR data structures only when the resources are written out to disk. The release R5 of FHIR changed terminological resources in two domains. First, all resources have additional metadata fields, such as *editor* or *reviewer*. As those are within the responsibility area of the standard FSH code **(DG2)**, the use of R5-specific metadata declarations requires the user to switch to the R5 processing mode. Otherwise, their use would result in validation errors, as they are unknown to the R4B validator.

In the area of resource content, minor changes present for CS and VS, which are handled by the proxy-class approach. However, CM has undergone a fundamental redesign which makes a version-agnostic implementation extremely challenging. Most importantly, the R5 CM requires a data element *relationship* which corresponds to the *equivalence* attribute in R4B. This attribute needs to be provided for each mapping entry and states the correspondence between the source and target concepts within the specified code system. The mandatory *CodeSystem* for stating this relationship was changed substantially, such that mapping these codes is a problematic undertaking that BabelFSH will not attempt automatically.

### Resource validation

To ensure that the generated resources are compliant with the specification, a comprehensive validation strategy has been implemented (**DG5**). The HAPI FHIR [40] library is used to prevent the generation of invalid output resources by our grammar-based FSH rule interpreter, which operates independent of the FHIR resource definition, and can thus not catch such errors on its own.

Moreover, users are provided an additional layer of validation through the integration of the FHIR validation engine that is maintained as a core infrastructural component by HL7 International in conjunction with the HAPI FHIR developers [41]. This additional validation step is opt-in and errors in this validation will not result in application termination, as many messages from this validation pipeline will be false positives. However, the validator will catch incorrect usage of profiles, missing elements etc. that might not be caught by the HAPI FHIR pipeline, so that the developer can rapidly iterate over the resource definition in their BabelFSH source files.

### Concept validation strategy

Both the correctness and the performance **(DG6)** of the proposed tool need to be assessed. As a point of comparison, the existing SUSHI reference compiler can be used: by generating standard FSH code for a suitable code system and compiling that with SUSHI both dimensions can be evaluated.

**Fig. 3:**
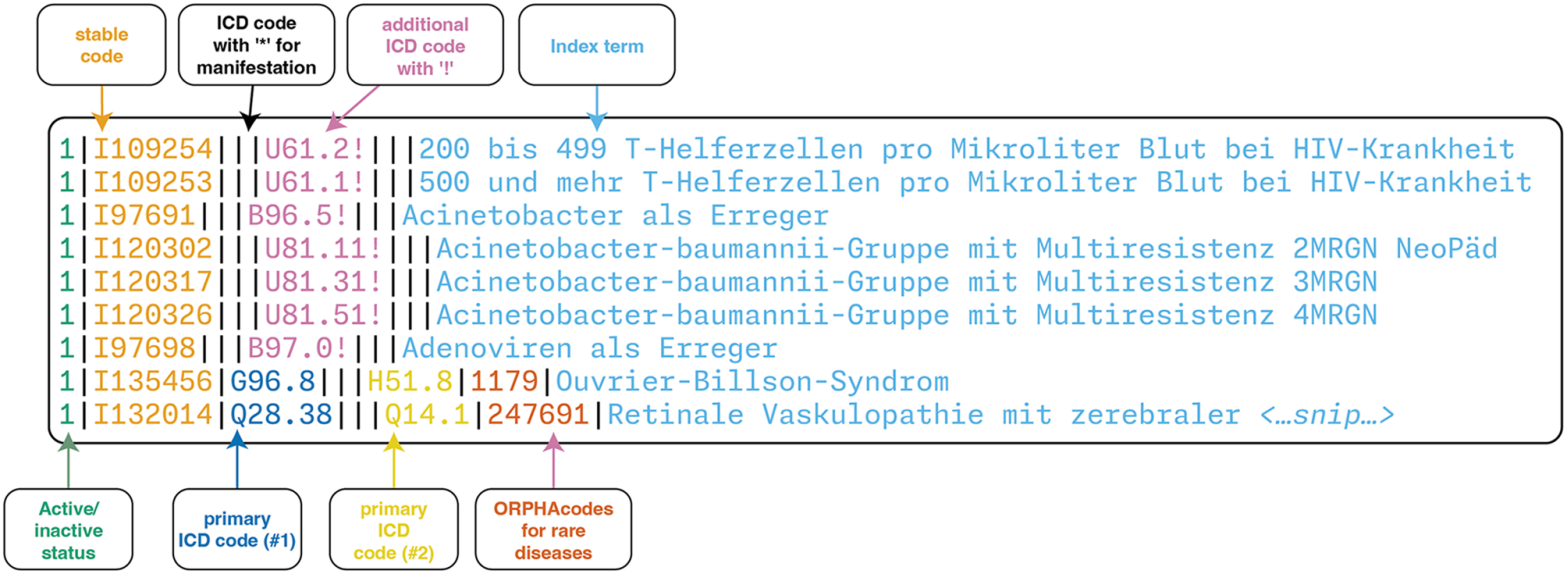
Excerpt from the Alpha-ID-SE 2025 source file, from [42]. The columnar format maps index terms (expressions from clinical practice) to a stable code (second column). Every concept is mapped to a selection of ICD-10-GM classes to classify the index term in the diagnosis classification. The ORPHAcode is an additional identifier for the identification of rare diseases

The German Alpha-ID-SE code system [42] is used for comparison. This artifact is an alphabetical index to the morbidity classification ICD-10-GM [43], with Alpha-ID-SE containing many more entries (2025 version: 90399) than ICD-10-GM (2025 version: 17089). For the more differentiated text entries below the ICD-10 code level, ORPHAcodes for rare diseases are added where possible [31]. The resource is distributed as a pipe-separated plain-text file (*source file)*, making the conversion to SUSHI straightforward. Each row of the file defines a single concept and several additional properties for these concepts. An excerpt of this file is shown in Fig. 3.

Using this file, the code system can be rendered as a standard FSH file using simple scripts. At the header of the output file, some metadata declarations are required, and from the source file, every line in that file will result in several lines of FSH code that declare not only the concept, but also the properties defined by Alpha-ID-SE. An equivalent BabelFSH file can be generated from the same template, where the metadata is declared identically (consistent with **DG1**, all FSH declarations should be correctly implemented by BabelFSH). By adding a BabelFSH plugin comment to that declaration, the content is then pulled out of the FSH declarations into the plugin architecture by providing the needed plugin arguments in the comment. From these two files, two JSON representations of the Alpha-ID-SE code system can be generated using SUSHI and BabelFSH, while the runtime of each approach is timed using command line tools.

Assessing the completeness of the generation is thus straightforward: if the number of concepts generated from the FSH and BabelFSH files are identical to the number of rows in the source file, the generation is complete. However, validating the completeness of the entire method is difficult to generalize, as that greatly depends on the plugin. Validating the completeness of each plugin thus needs to be done by the plugin authors on a case-by-case basis, As the source code for each plugin will generally be quite compact, they will be easily testable and debuggable.

### Usability evaluation

To study the usability of the system, a usability study has been performed. To this end, participants were asked to perform a task and subsequently fill a standardized survey. As recommended by Sauro and Lewis [44, p. 186], the System Usability Scale (SUS score) [45] is used as the post-study questionnaire. The study was conducted on-line using the LimeSurvey platform [46] hosted by the University of Luebeck. No personally identifiable data was directly collected in the survey. The full questionnaire and the accompanying resources are publicly archived in Zenodo (see the *Availability of data and material* section).

Respondents were recruited from the Medical Informatics Initiative and the Technical Committees for terminology and FHIR of the German national HL7 affiliate, HL7 Germany. As such, they were assumed to be familiar with the FHIR standard in general and profiling and the use of FSH/SUSHI in particular. Before the standardized task, respondents were asked to rate their familiarity with FSH and command-line tools in general. The survey then briefly explained the BabelFSH concept in general. Next, the participants were guided through setting up BabelFSH on their computers. Through a Git repository, they were provided with the following resources:

- a wrapper script to download the BabelFSH application from a web storage site if needed for Windows and Linux/macOS,
- the files needed for working through the task,
- a printable version of the setup steps as a reference,
- and a solution of the task, if needed.

The task required users to convert a small tabular file with 33 concepts. The code system was pseudo-randomly generated by a large language model (Claude Sonnet 4), as it is only intended as an illustrative example of the possibilities enabled by the BabelFSH concept and is not intended for actual use. Users were asked to write BabelFSH source files to convert this CSV-style file into a FHIR CS, mirroring the expected output that was provided to them as closely as possible. The conciseness of the input files ensures that the output file will also be concise, such that the respondents should be able to identify all required fields while iterating on their FSH code.

The columnar format of the input file featured a “CODE” column, a “DISPLAY” column, and two properties. First, an “INACTIVE” column with the answers “YES” and “NO” flags concepts as deprecated. The “PARENT” column directly maps to the parent property in FHIR CS. An excerpt of this CSV file and the resulting FHIR CodeSystem representation is available in Fig. 1 (bottom left and right).

After working through this task, users answered the ten-item SUS score via a five-point Likert scale (*strongly disagree* / *disagree* / *neither agree nor disagree* / *agree* / *strongly agree).* Following the research of Sauro and Lewis [47, 44 p. 206–11], the alternate version of the SUS score was used where all items are positively phrased, rather than alternating positive and negative items [45]. The positive phrasing results in less cognitive effort on the participants, since higher scores will always correspond to better usability [44, p. 208].

Finally, the respondents had the opportunity to report any difficulties they encountered alongside resolution strategies, as well as further feedback about the tool and about the survey.

## Results

### System implementation

**Fig. 4:**
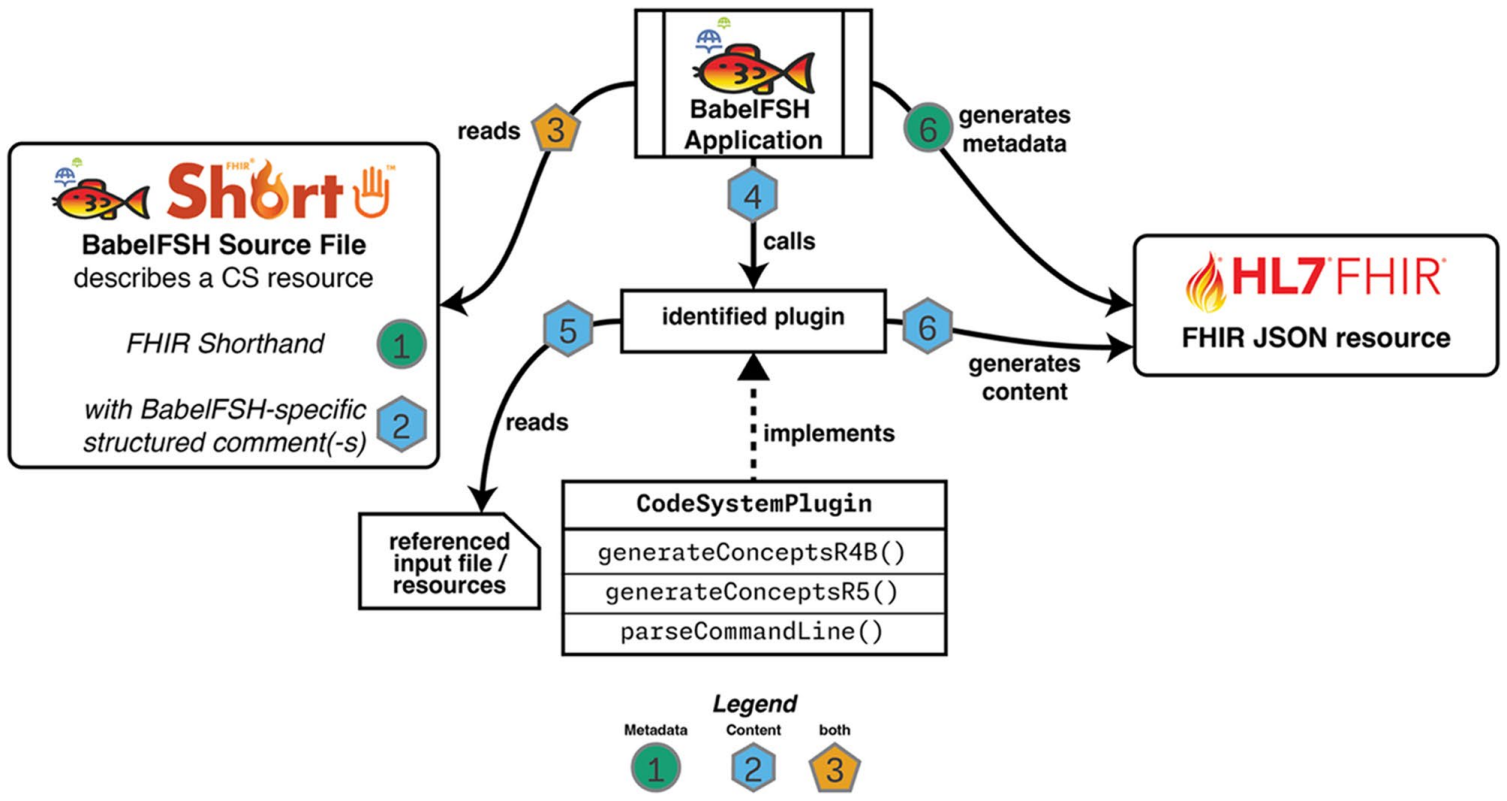
High-level concept & implementation of the BabelFSH approach. The input files provide metadata definitions through FSH code and content through structured comments as required by the BabelFSH application

The resulting system is shown in Fig. 4. First, BabelFSH identifies the input files using filename matchers, with all files needing the extensions “.babel.fsh” or “.babelfsh.fsh” to be considered by the tool. This is to ensure a clear delineation between aspects better served by SUSHI and those to be solved by BabelFSH. The BabelFSH approach is implemented through these steps:

1. Users specify the metadata of terminology resources (e.g., a *CodeSystem*) through FSH rules.
2. BabelFSH-specific comments provide the definition for the content conversion process using a command line syntax.
3. The source files are read and parsed by the BabelFSH app.
4. The selected plugin is called with the provided command line arguments, which parses and validates these arguments.
5. Any referenced input resources are read in the plugin’s code and transformed to content items by the plugin.
6. The metadata through the FSH code (green) and content through the plugin (teal) are combined into a FHIR JSON resource and written to disk.

### Evaluation

For comparing the performance of our approach with the state-of-the art SUSHI compiler, FSH code was generated from the Alpha-ID-SE sources in version 2025. The implementation of the FSH conversion was accomplished through a Python script that reads the file line by line and uses string templates to generate FSH statements for each concept.

The approximately 90 400 input lines (4825 KiB) thus balloon to more than 500 000 lines (+ 453%) of FSH code (21 492 KiB, + 345%) to not only define the concepts and displays, but also the metadata for each concept available in the resource. Converting the sources on the test computer, an Apple MacBook Pro with a M4 Pro processor and 48 GB of RAM, was done in less than one second.

**Fig. 5:**
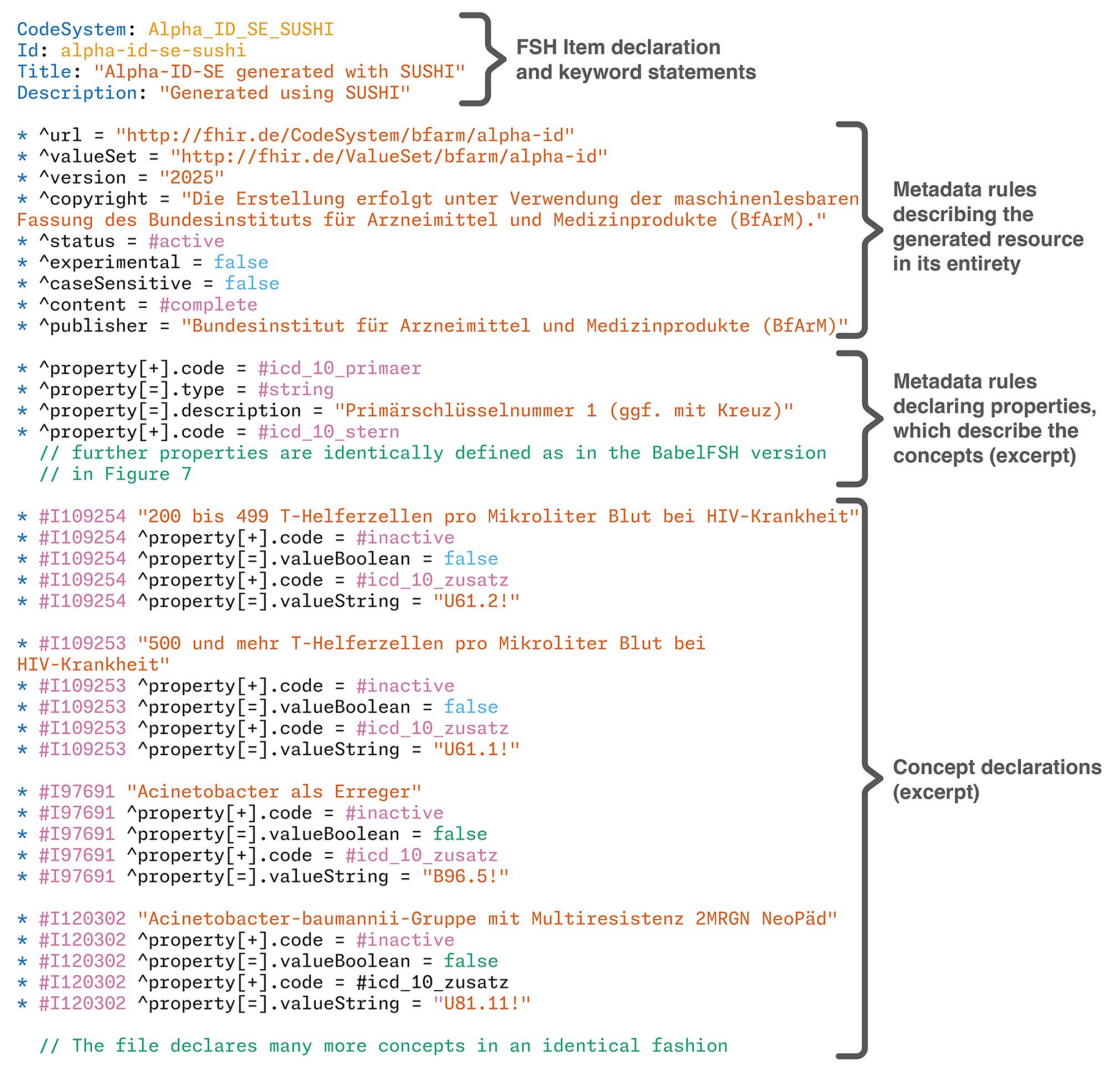
Annotated excerpt from the generated FSH file for validation using SUSHI

**Fig. 6:**
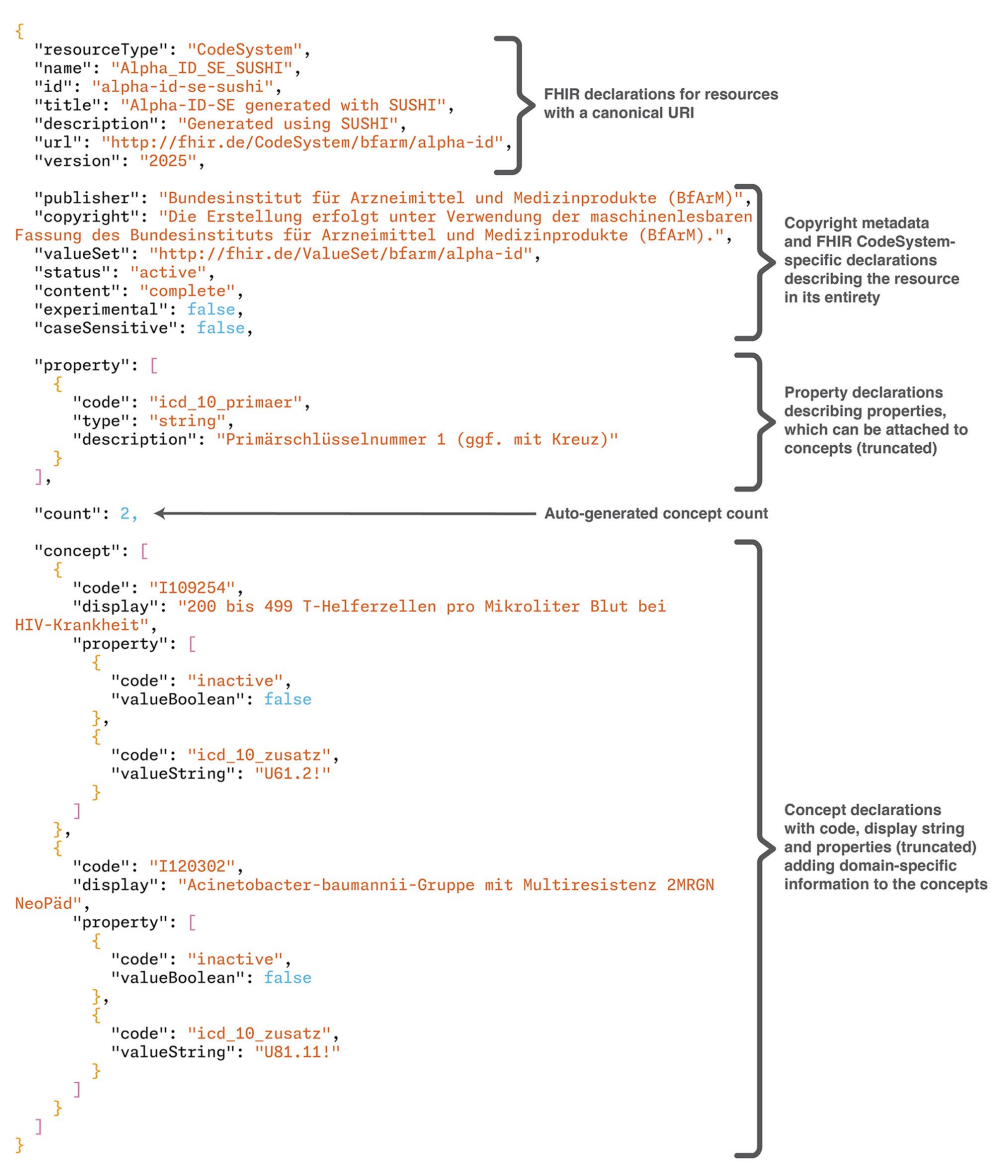
Annotated and truncated CodeSystem generated from the FSH code in Fig. 5

However, it was apparent that SUSHI struggles with the generation of content from this extremely large file. The *gnomon* tool [48] was used to time that SUSHI version 3.14.0 took approximately 16 s just to read in the file, and generating the single FHIR resource from this file took an additional 812 s. All in all, the command terminated after 833 s or 13.9 min wall clock time, with the laptop running in high-power mode and being otherwise idle. A short excerpt of the generated FSH file is available in Fig. 5, and the resulting FHIR JSON representation in Fig. 6.

**Fig. 7:**
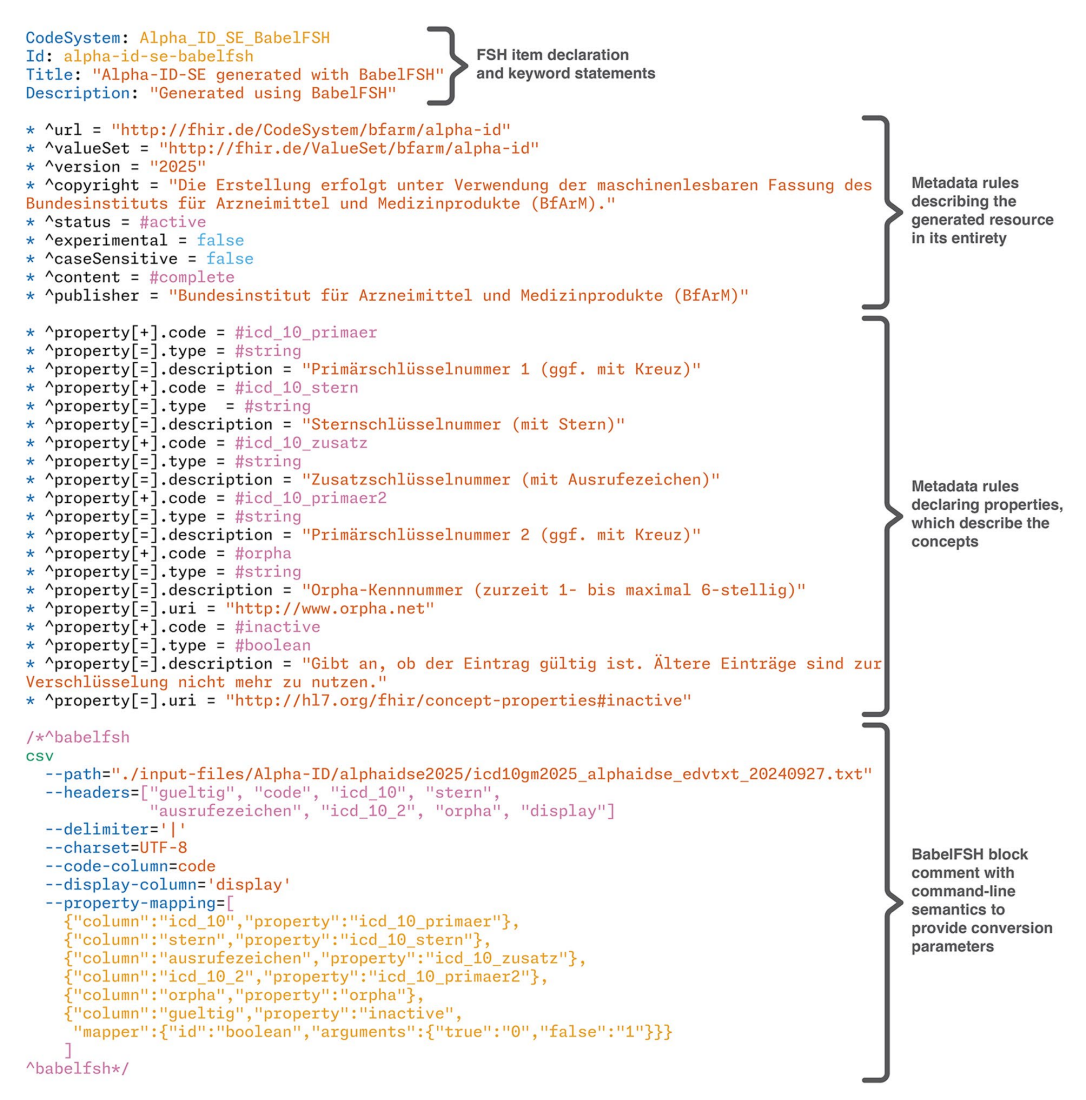
Complete BabelFSH source file for conversion of Alpha-ID-SE 2025

Comparing this with the BabelFSH approach, a single version of Alpha-ID-SE was defined in less than 60 lines of FSH code with the metadata of the resource being identical to the standard FSH version. Converting this resource to FHIR JSON took less than 3 s in total. The full BabelFSH source file is available in Fig. 7.

As a further point of comparison, defining all available versions of Alpha-ID-SE starting in 2015, when the resource first became available, required less than 160 lines of code (including whitespace and comments) and compiled in 16.1 s (with the supplemental validation enabled), demonstrating the power of FSH in terms of definition re-use. Using parametrized rulesets, the declaration of the FSH item for each version only requires five lines of self-explanatory code.

For the example of Alpha-ID-SE, it could be verified that the generated resources are indeed semantically identical: all metadata is present, all concepts from the sources are defined, and all properties are mapped to FHIR properties. By formatting the resources using JSON tooling and comparing them using the standard GNU *diff* tool, this equivalence could be rigidly asserted.

All resources for this validation are available in the BabelFSH source code repository.

### Usability study

In total, eight respondents have completed the usability survey, while it was started by 11 more people. Due to the study design inherent with post-task usability studies, completion of the survey was likely not possible for some of the participants due to other commitments. Due the small number of participants, the study will only be evaluated qualitatively in this paper.

The full study result export, the study definition in the LimeSurvey format and more graphical evaluations of the results (using the R programming language, with full source code) is available in the Zenodo archive (see below). All participants have worked with the FHIR standard and are at least moderately comfortable using command line interfaces. Not all participants were familiar with SUSHI and FSH.

With regards to the SUS score, participants were asked to grade the system on a five-point Likert scale after working through the task using these ten aspects [44, 47]:

- **Q01**: I think that I would like to **use** the tool **frequently**.
- **Q02**: I found the tool to be **simple**.
- **Q03**: I thought the tool was **easy to use**.
- **Q04**: I think that I could use the tool **without** the **support** of a technical person.
- **Q05**: I found the various functions in the tool were well **integrated**.
- **Q06**: I thought there was a lot of **consistency** in the tool.
- **Q07**: I would imagine that most people would **learn** to use the tool very **quickly**.
- **Q08**: I found the tool very **intuitive**.
- **Q09**: I felt very **confident** using the tool.
- **Q10**: I could use the tool without having to **learn anything new**.

**Fig. 8:**
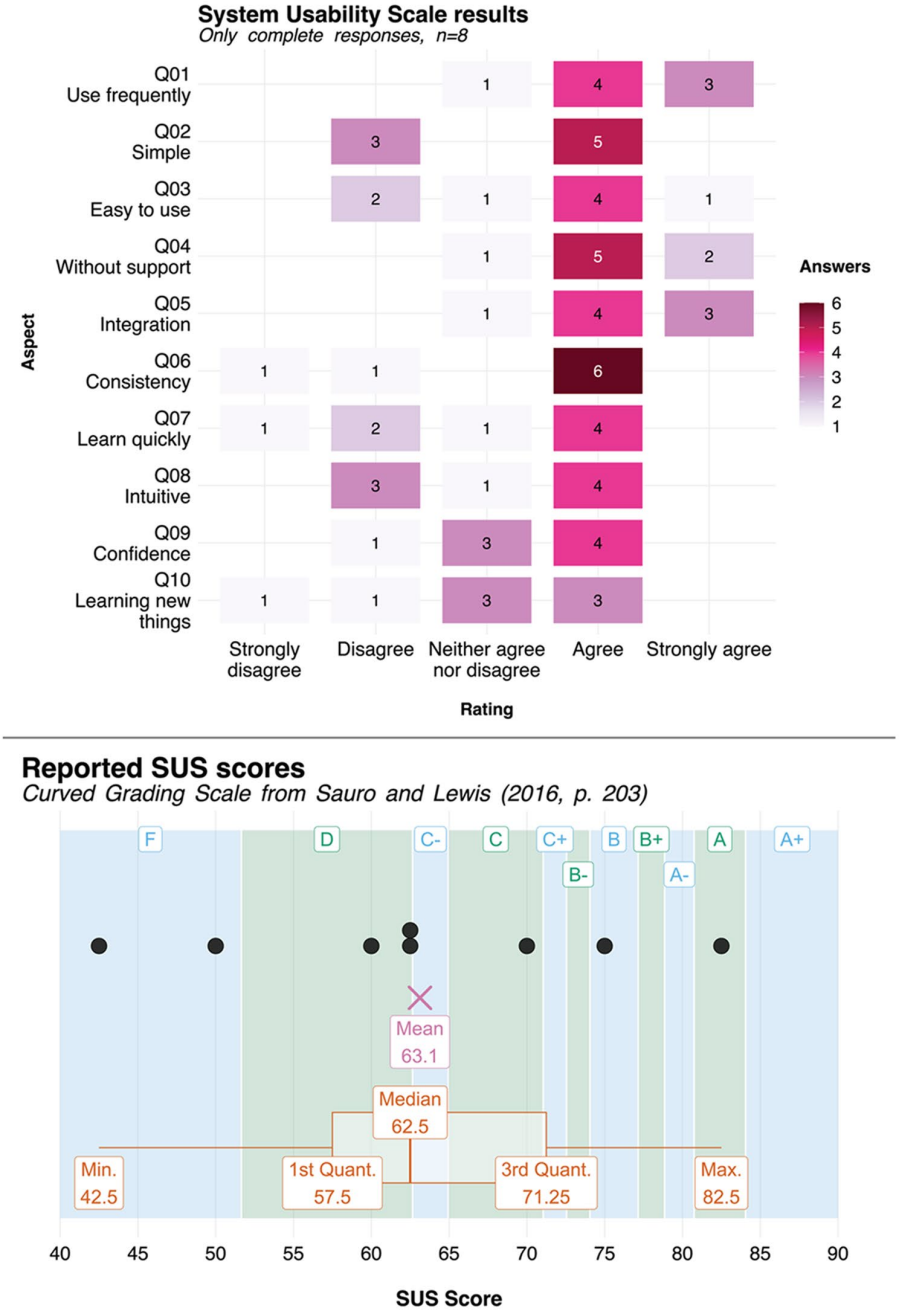
Usability study results. Top: raw item scores; bottom: raw SUS scores and descriptive statistics (mean, median, minimum and maximum values, first and third quantile). The blue and green shades represent the Sauro-Lewis grade ranges [44, p. 204]. They do not define an “E” grade

The individual results are shown in Fig. 8 (top). Bold-face highlights represent the keywords used in place of the full item text in the analysis.

The SUS score is calculated from the raw items by mapping the answers to the range from 0 to 4 (strongly disagree = 0). These values are summed, and the sum is mapped to the range from 0 to 100 by multiplying with 2.5. The analysis of the overall SUS score is shown in the bottom of Fig. 8. The grading scale by Sauro and Lewis is shown in blue and green. Black bullets represent the raw answers.

### System in use

As has been motivated in the background section, BabelFSH was conceived of in the context of the requirements for the provision of a research FHIR terminology server within the MII, having a national scope. Using the BabelFSH tool, the initial efforts and the continued maintenance of diverse resources accessible to these services has been streamlined. As of writing, resources defined by the German BfArM (ICD-10-GM, OPS, ICD-O-3, ASK), the German adaptation of ATC, several important OWL ontologies including the Gene Ontology or the Human Phenotype Ontology, the EDQM Standard Terms database, the HGNC gene names database and others are generated and distributed using BabelFSH (cf. Table 1 for explanations for each terminology thus generated).

**Fig. 9:**
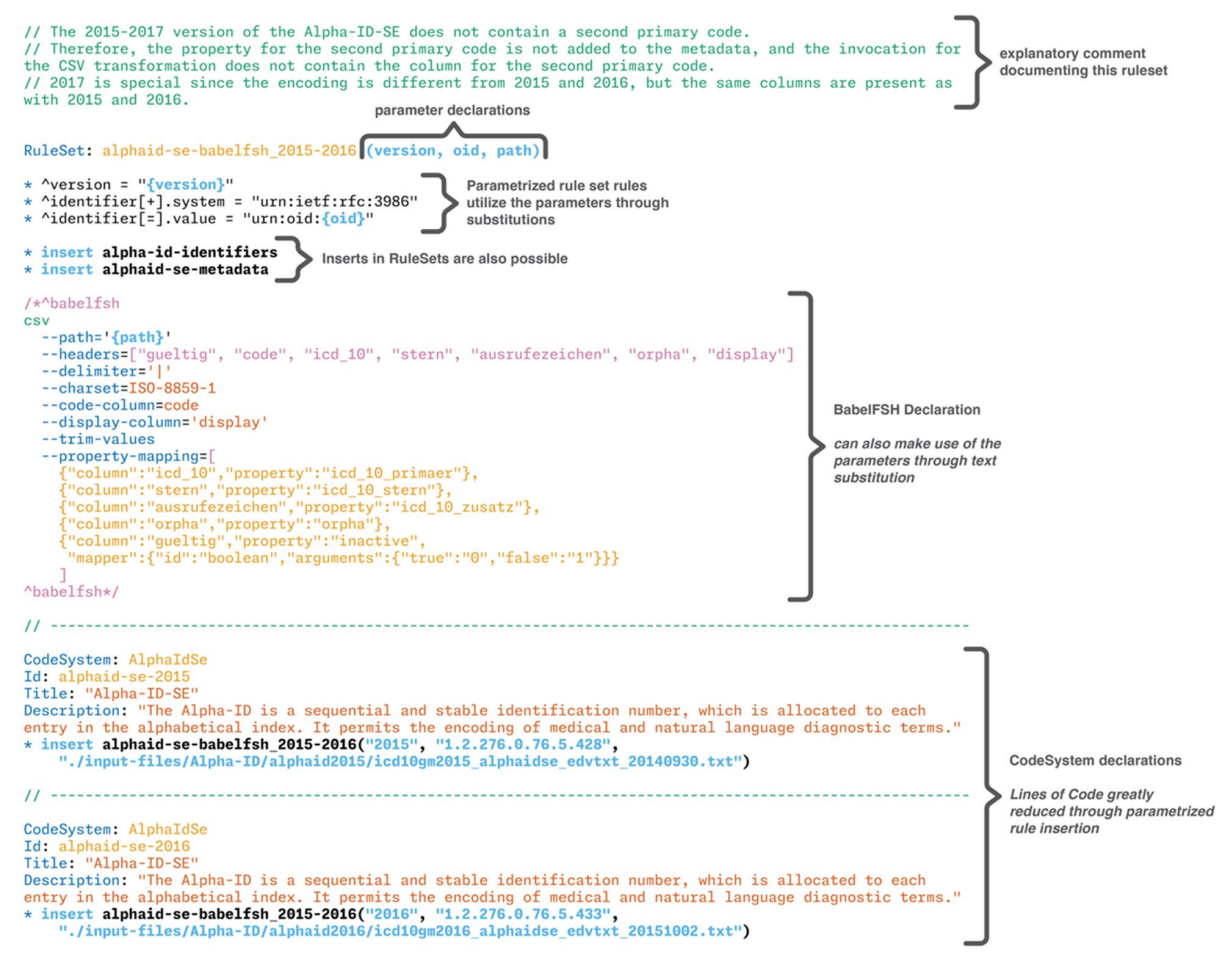
RuleSet re-use in the conversion of Alpha-ID-SE. This accommodates version differences over time when providing multiple versions of a resource to consumers. RuleSets can in turn include RuleSets, allowing for clear separation of concerns and readable FSH declarations

In this use of the developed system, the facility for rule re-use is especially helpful to rapidly and consistently generate multiple versions of some resources. An example is shown in Fig. 9, taken from the repository for Alpha-ID-SE generation [49]. As the distribution format for Alpha-ID-SE has changed slightly over time, the re-use of declarations depending on the versions illustrates the differences clearly. In practice, the source file currently contains three version-specific RuleSet, while the core metadata is defined in a single RuleSet (*alpha-id-se-metadata*). Parameters are substituted at run-time by the BabelFSH system according to the FSH specification, allowing for very compact FSH items, providing a clear separation of concerns and reducing the possibility of copy-paste errors for users.

Moreover, besides the direct sharing of resources, the system could be used to provide access to FHIR representations of resources that must be licensed for machine-readable distribution. In particular, the Medical Dictionary for Regulatory Activities (MedDRA) terminology [50] can only be distributed to licensees, which the TS can currently not enforce. By distributing the BabelFSH source files, however, all parties that have a MedDRA subscription and license can generate identical representations of this input, making the distribution of the FHIR resource to those parties mostly redundant.

## Discussion

All design goals previously laid out are believed to be suitably addressed. A complete assessment and a summary of the strategy for achieving each goal is stated in Table 2. While the development guided by the design goals did not follow any strict software engineering methodology, their definition ahead of the implementation was extremely helpful in guiding the development process. The design and implementation and performance of the system was positively discussed within the FHIR community, both during in-person meetings of the German FHIR standardization community as well as on-line and during the 2025 edition of the developer conference *FHIR DevDays* with international members of the community.

**Table 2:** Design goals and implementation in the BabelFSH application

Design Goal	Implementation
DG1—Compatibility with SUSHI	BabelFSH supports a strict subset of the FSH specification, focusing on terminology resources. All valid BabelFSH source files are thus also valid FSH files.
DG2—Metadata in FSH	The tool supports a clear separation of concerns through the plugin architecture.
DG3—Extensibility	The provision of new plugins requires only the implementation of a very simple API.
DG4—Simple API and Self-Documentation	Based on the provided API, all plugins must be documented by the plugin arguments and further help texts, such that automatic help can be generated.
DG5—Validation	All FSH rules are validated for compliance with the FHIR specification through established validation tooling integrated into the software. Additional validation rules are enforced in the content generation pipeline.
DG6—Performance	BabelFSH has proven to be performant and scalable.
DG7—FHIR Version support	FHIR versions R4B and R5 are supported through a mode switch, and further versions could be added to the API as they are released.
DG8—Open Source	BabelFSH is freely available under the Open Source Initiative-approved Apache 2.0 license.

While the turnout rate of the usability study was very low, the SUS score indicates that the system is usable as intended. With a mean SUS of 63.1 and a median of 62.5, the system falls in the Sauro-Lewis grade range [44, p. 204] from *D* to *C-*. While this score places BabelFSH only around the 40th percentile, it is nevertheless adequate for a system that is inherently quite specialized and complicated. Moreover, the authors are so far not aware of any usability study that was performed with regards to FHIR developer-centric tooling, so it not possible to compare BabelFSH’s usability to any other specialized FHIR tool.

Moreover, the per-item analysis of the responses is quite positive: 52 of the 80 answers were assigned on the positive part of the scale, 12 were neutral and 16 were negative. While this might be an indication of acquiescence bias in the results (users might be more inclined to answer positively), the degree to which this is a factor that cannot be assessed in this study. Regardless, the study supports the hypothesis that (with training and experience), users can use this specialized tool appropriately and independently.

The highly constructive free-text responses to the survey also point out the lack of documentation in some areas and unintuitive command line arguments in the BabelFSH comments, which can this feed back into the development process of the tool and accompanying resources. No respondent has given an indication that the overall approach and implementation of the tool is flawed, and the addition of high-quality documentation will certainly improve usability of the overall systems. The responses also indicate that some of the problems the users encountered might have been due to the wrapper script provided in the evaluation. This wrapper was specifically written to support the evaluation and is thus not a substantial part of the system.

The most pressing limitation of our system currently is the number and selection of available plugins. As our system originates in the context of a well-defined project, our own efforts in plugin development in particular center around the needs for this project. Due to the open-source nature and elegant simplicity of the approach, the authors believe that the broader FHIR community will recognize the potential of this tool, and define their own plugins as needed. The application design does also lend itself to the creation of advanced plugins, such as the implementation of a database connector that would be able to connect, using standard database access layers, to legacy systems that define internal catalogs, which could then be integrated into FHIR resources as needed.

In terms of the API, the CS generation API can currently be considered mature, since it has proven itself for several quite different plugins now. That for CM is likely stable, but VS generation has not been considered extensively so far. As there are two ways of defining VS that could be defined, this API requires careful consideration concerning the intended scope of the generation. First, the intensional approach defines a VS based on rules that are evaluated by the terminological services, e.g. all concepts that are children of a specific concept. Second, in extensional definitions, the included concepts are listed directly. Especially for intensionally-defined VS, the SUSHI implementation is already adequate, while extensional VS can, depending on their size, present the same (performance) limitations that SUSHI presents for CS resources. Thus, further efforts will be concentrated around providing a facility to generating these extensional VS.

A further limitation of the current implementation is our input validation. BabelFSH currently has no concept about the FHIR metadata structures and applies the rules as they are written. If there is a typo or other error in the declarations, this will be caught by the HAPI validator [40] instead. Complex data elements such as *CodeableConcept* however are thus challenging to validate, resulting in imprecise source code coordinates in the error messages. Since these complex elements cannot be validated line-by-line, the messages will only indicate a block of multiple lines where the error might have occurred. However, as the target group of BabelFSH are advanced FHIR implementers, this level of error-checking is deemed adequate, since the messages generated in this way are nevertheless quite descriptive. Errors resulting in incorrect usage of the command line arguments also surfaced in the usability study, leading to user frustration.

An interesting consideration for the future could be the integration of this tool into the reference standard SUSHI compiler and, potentially, the integration of new language elements into the FSH language definition. In this way, the functionality provided by BabelFSH could be integrated with the standard build tools that are now established in the FHIR community for IG creation. However, the intended user groups of BabelFSH and SUSHI are not always the same people. SUSHI is geared more towards IG creators, while BabelFSH is aimed at those providing FHIR terminology resources or services. While there is a degree of overlap between those user groups, it is rare for FHIR IGs to be responsible for the maintenance and distribution of larger-scale terminologies. The narrow focus of BabelFSH and the inherent constraints of the FSH implementation can allow for quicker iteration, cleaner code and faster learning for terminology conversion compared to a SUSHI-native implementation.

Moreover, the command-line interface of the BabelFSH app itself still allows integration into automation processes including Continuous Integration (CI) pipelines that are now common in the development of IGs. Standard IGs built using SUSHI include an auto-generated script (*_genonce.sh/_genonce.bat*) which uses the SUSHI and the IG Publisher tool to build the resources and the human-readable implementation guide. CI build pipelines can in turn call this script to generate the sources for publication on the internet. In principle, the BabelFSH command line calls could be integrated into this build pipeline via the *_genonce* script to automatically generate terminology sources alongside the other FHIR resources. However, the scripts can also be updated automatically and would have to include facilities for BabelFSH integration from the upstream distribution. A more robust process will, for the time being, integrate BabelFSH into the generation process using a distinct step in the CI pipeline before the IG build is run.

In this regard, the performance of the implemented system is also beneficial, as long runtimes of the conversion processes could be an obstacle to the profilers due to the time and cost penalties incurred by a longer runtime during IG builds on the CI infrastructure. Through caching, terminology resources could also be re-generated in the CI only if an update is needed.

Regarding the integration of this plugin architecture into the FSH language specification via a new language element instead of the block comments that are currently used, this document has now reached a level of maturity that makes most rules normative, disallowing changes that are not backwards compatible. While adding new language elements is not a breaking change, there are some constraints in the BabelFSH implementation that may not be compatible with the specification as written, such that integration would require very careful consideration.

Comparing the developed system to TermX [29] in particular, it must be noted that the scope of the developed application is inherently different to the end-to-end approach offered by TermX. While TermX can also import terminologies through use-case specific adapters, these must be implemented for each terminology format. BabelFSH can thus complement the facilities of TermX through offering an alternative approach to importing external terminology resources and is orthogonal to the existing efforts. In principle, the TermX application could likely be refactored such that all terminology imports are realized internally through the BabelFSH application. Additionally, as all FHIR terminology tools can generally import FHIR resources natively, they can directly consume data generated by the BabelFSH tool without source code changes.

Lastly, for FHIR terminology services to generate a benefit to implementers and users, the maintenance and content delivery of these systems needs to be integrated with the requirements of all stakeholders. Since FHIR TS are generally only deployed within a broader infrastructure, the content delivery of such a service needs to be carried out in lockstep with other changes in the infrastructure. A tool like BabelFSH can aid in that area, by both allowing fast reaction to new or shifted requirements, such as providing new resources, and the maintenance of such resources in the future, but is only one tool towards professionalized TS maintenance. Within the SU-TermServ project, providing such a service with a broad scope, BabelFSH has become an important tool, but only develops its potential through the integration into a comprehensive strategy and tooling infrastructure.

## Conclusions

In this work, a novel, powerful and open-source approach to making heterogeneous sources of terminological knowledge broadly accessible as FHIR resources has been presented. In this way, BabelFSH is an important tool towards the adoption of comprehensive HL7 FHIR terminological services, and thus greater (semantic) interoperability in general. It is furthermore orthogonal to existing approaches to and tools for FHIR terminology conversion: the core logic for content generations is often cleanly separable from the (often hard-coded) generation of the needed metadata, such that the generation part can be easily integrated into the BabelFSH tool.

Usage of the BabelFSH tool and uptake by the community can ensure the availability of required terminological resources through FHIR terminology services, aiding the adoption of the HL7 FHIR standard overall through stronger semantic interoperability of healthcare data. The tool has the potential to be adopted by terminology authors to streamline the provision of authoritative FHIR representations of their terminologies in the first place, such that community efforts to generate such artifacts become unnecessary.

## Data Availability

Project name: BabelFSH Project home page: https://gitlab.com/mii-termserv/babelfsh; web documentation at https://su-termserv.gitbook.io/babelfsh Archived version: Source code: 10.5281/zenodo.15755172. Usability study: 10.5281/zenodo.17207837 Operating system: Platform independent Programming language: Kotlin (primarily), Java Other requirements: Java 18 or higher, Gradle License: Apache License 2.0 Any restrictions to use by non-academics: none apply.

## Footnotes

1 A FHIR representation of this resource has now been put forward by the BfArM. At the time BabelFSH was conceptualized, this resource did not yet exist, so that a conversion of the ClaML representation was quite relevant at the time. As of writing, this FHIR representation is not available for all terminologies in all versions maintained by the BfArM.

## Declarations

### Authors’ information

As part of the Medical Informatics Initiative, all authors are responsible for the provision and development of FHIR-based terminological services to the MII across Germany, and have substantial experience in working with medical terminologies and FHIR terminological services. They are active in the development of the FHIR standard and national adaptations through HL7 Germany, and in the working groups of the German Association for Medical Informatics, Biometry and Epidemiology (GMDS).

## Abbreviations

API: Application Programming Interface
BfArM: *Bundesinstitut für Arzneimittel und Medizinprodukte* (German Federal Institute for Drugs and Medical Devices)
ClaML: Classification Markup Language
CI: Continuous Integration
CM: ConceptMap (a concrete FHIR resource)
CS: CodeSystem (a concrete FHIR resource)
CSV: Comma Separated Values, interpreted to include all kinds of files with delimiters, including commas, semicolons, pipe characters, tab stops, etc
DSL: Domain-Specific Language
DSTU1/DSTU2: Draft Standard for Trial Use Release 1/2 (a version of the HL7 FHIR standard)
EDQM: European Directorate for the Quality of Medicines & HealthCare
EHDS: European Health Data Space
FSH: FHIR Shorthand
HL7 FHIR: Health Level 7 Fast Healthcare Interoperability Resources
IG: Implementation Guide
MedDRA: Medical Dictionary for Regulatory Activities
MII: Medical Informatics Initiative
NUM: Network University Medicine
OWL: Web Ontology Language
R4/R4B/R5: HL7 FHIR Release 4/Release 4B/Release 5
STU3: Standard for Trial Use Release 3 (a version of the HL7 FHIR standard)
SUS: System Usability Scale
SU-TermServ: Service Unit Terminological Services
SUSHI: SUSHI Unshortens Short Hand Inputs (the reference compiler for FSH)
TS: Terminological services
VS: ValueSet (a concrete FHIR resource)
